# Hypobaric Resilience of Matrix Polysaccharide Biosynthesis in Plants

**DOI:** 10.1002/pld3.70190

**Published:** 2026-10-04

**Authors:** Hunter F. Strickland, Talia Jacobson, Grace Smith, Cătălin Voiniciuc, Andrew C. Schuerger, Anna‐Lisa Paul, Robert Ferl

**Affiliations:** ^1^ Plant Molecular and Cellular Biology Program University of Florida Gainesville Florida USA; ^2^ Department of Horticultural Sciences University of Florida Gainesville Florida USA; ^3^ Institute of Food and Agricultural Sciences University of Florida Gainesville Florida USA; ^4^ Department of Plant Pathology University of Florida Gainesville Florida USA; ^5^ Interdisciplinary Center for Biotechnology Research University of Florida Gainesville Florida USA

**Keywords:** analog environment, cell wall matrix, hypobaric, low pressure, polysaccharide

## Abstract

Space habitats and transit vehicle interiors are proposed to operate at less than one Earth atmosphere (hypobaria) to minimize engineering costs in space‐based bioregenerative life support systems (BLSS). Understanding how plants are affected by lower‐pressure spaceflight environments and associated stressors will be vital for their use in life support within BLSS habitats. Whereas much is known about plant physiology in hypobaria, including the differential expression of genes involved in cell wall metabolism, little is known about the effects of hypobaria on plant cell wall matrix polysaccharide composition. The current study characterized the cell wall polysaccharide compositions of two Arabidopsis ecotypes and two mutants (*sweet11* and *pdc1*) affecting hypobaria‐responsive genes. Seedlings were cultivated in four atmospheric pressure environments (33, 50, 75, and 98 kPa) for 3 days prior to cell wall profiling using high‐performance anion‐exchange chromatography with pulsed amperometric detection (HPAEC‐PAD). Cell wall matrix polysaccharides were not consistently altered in three independent experiments at the atmospheric pressures listed above. These results reveal the resilience of plant cell walls to hypobaric stress and provide encouraging results for plants within low‐pressure space habitats.

## 1 Introduction

Future space transit vehicles and habitats are proposed to operate at low atmospheric pressures (hypobaria) to help alleviate engineering constraints, better facilitate extravehicular activities (EVAs), and conserve resources (Rygalov et al. 2002; Corey et al. 2002; Lange et al. 2005). Proposed long‐term missions have also envisioned plant‐based bioregenerative life support systems (BLSS) as crucial components (De Micco et al. 2023; Wheeler 2010). Thus, understanding how plants grow in and respond to hypobaric environments will be required for the success of future exploration endeavors. Plants evolved to colonize terrestrial environments, which range in atmospheric pressures from sea level (101.3 kPa) to the highest terrestrial point on Earth—the peak of Mount Everest at 8847 m where pressure is 33 kPa (Junyong et al. 2010). However, most plants of agronomic use to humans are more accustomed to atmospheric pressures between sea level and about 70 kPa.

The reduced atmospheric pressure of high‐altitude terrestrial environments is also hypoxic, due to the reduction in the partial pressure of oxygen as the total atmospheric pressure decreases. Altitude plays a role in the limits of plant habitation in natural environments, but the limitations are primarily due to how terrestrial altitudes create a harsh combination of low and fluctuating temperatures, drought, hypoxia, and UV radiation (Dentant 2018). High‐altitude plants have developed a variety of adaptive strategies to cope with the extremes, but it appears that hypobaria itself is less of a constraint. Terrestrial plant habitation appears to be limited to around 6000 m–where the atmospheric pressure is 47 kPa (Angel et al. 2016). When environmental stress eases during warmer and wetter periods in high‐altitude habitats, plant species such as *Draba altaica* will expand their range to higher altitudes with the accompanying lower pressures (Angel et al. 2016). Whereas *D. altaica* may be adapted to withstand hypobaria, the fitness of, and molecular consequences on, low‐altitude plants at hypobaric pressures remains unclear.

Irrespective of the limitations for plant growth in regions of terrestrial hypobaria, hypobaric environments below 50 kPa are outside of the evolutionary experience of terrestrial plants and present a novel stress, even when water, oxygen, and temperature are not limiting (Paul and Ferl 2006). Hypobaric environments are known to cause detectable changes in plant physiology and gene expression. Two physiological consequences observed in multiple species are increased transpiration and gas diffusion rates as the atmospheric pressure decreases (Goto et al. 1996; Gohil et al. 2011; Richards et al. 2006).

Plants have shown they respond well under mild hypobaric conditions when examining plant health metrics like plant biomass, height, production, and photosynthesis rates (Gohil et al. 2011; Rajapakse et al. 2009; Ishigami and Goto 2008). Plant biomass accumulation remains stable, and photosynthesis can even increase under mild hypobaric conditions (Goto et al. 1996; Ishigami and Goto 2008). Genes associated with the plant cell wall are differentially expressed under hypobaric conditions ranging from 75 to 10 kPa (Paul et al. 2004; Paul et al. 2017; Zhou et al. 2017). Genes differentially expressed included both passive and active sugar transporters (e.g., SWEET11, Sugars Will Eventually be Exported Transporter 11), glycosyl hydrolases (e.g., XTH16, xyloglucan endo‐transglycosylase/hydrolase 16), and cell wall polysaccharide biosynthesis (GAUT7, galacturonosyltransferase 7) genes. Perturbations in these cell wall–related genes would support the hypothesis that cell walls are affected by hypobaric conditions.

Despite the physiological and transcriptomic data (cited above) suggesting plant cell wall modifications in hypobaric conditions, biochemical data to support these cell wall hypotheses have been lacking. To address this knowledge gap, *Arabidopsis thaliana* plants were exposed to mild (75 and 50 kPa) and severe (33 kPa) low‐pressure environments with subsequent cell wall polysaccharide composition analysis. The resilience, or the ability to maintain a state similar to a control or wild type, of Arabidopsis cell walls to hypobaric environments was then determined utilizing the cell wall profiling technique conducted herein, HPAEC‐PAD.

## 2 Materials and Methods

### 2.1 Plant Materials and Growth Conditions

The current study utilized four *A. thaliana* (Arabidopsis) accessions including Columbia (Col‐0), Wassilewskija (Ws), SALK_090204C (*pdc1*), and SALK_073269 (*sweet11*). The knockout lines *pdc1* and *sweet11* originated from the Col‐0 background and have been previously described (Gravot et al. 2016; Le Hir et al. 2015) and were obtained from the Arabidopsis Biological Resource Center (ABRC). Additional homozygosity verifications were conducted on both mutant lines with genotype‐specific primers generated using the Salk T‐DNA primer design tool (T‐DNA Primer Design). Specific primer sequences are provided in the supplemental (Table S1.1). Plants were grown in a vertical orientation under constant light for 10 days prior to the 3‐day atmospheric pressure treatment. The plants were grown at 23°C ± 0.8 for the duration of their growth and atmospheric pressure treatments. The seeds were sown aseptically on 100 mL of Phytagel media consistent with the methods described in Paul et al., with the addition of the fungicide benomyl (Paul et al. 2001) added to 5 ppm to the media in circular petri dishes (150 mm, Genesee Scientific, Morrisville, NC, USA). The harvested tissues were flash frozen using liquid nitrogen following dissection of the roots and shoots and kept at −80°C until monosaccharide analysis.

### 2.2 Pressure Treatments and Equipment

For the composition‐focused experiment three biological replicates were spread across three individual yet interconnected pressure chambers, which were connected to a single pressure controller (KNF Vacuum and Controller, Model #DC2/1K, Breisgau, Germany; Figure 1). For each atmospheric pressure, treatment plants from two plates were combined into one biological replicate due to the amount of tissue required for chromatography‐based analysis. The atmospheric pressure of the chambers was immediately taken to the target atmospheric pressure within 1–2 min. The pressure controller was set to 0.4–0.6 kPa below the target atmospheric pressure with a hysteresis of 1 kPa. During the hypobaric treatments, atmospheric composition was not artificially manipulated; therefore, reducing the total pressure also reduced the partial pressure of oxygen, creating a combined hypobaric–hypoxic environment. Consequently, the partial pressure of oxygen decreased to approximately 16, 10.7, and 7 kPa during the 75, 50, and 33 kPa treatments, respectively. Two of the chambers contained independent atmospheric pressure monitors which included a BAR20 (Dracal Technologies, Quebec, Canada) and an MSR datalogger (MSR, Seuzach, Switzerland). Each of the cylindrical pressure chambers was composed of a polycarbonate chamber with an interior volume of approximately 7500 cm^3^ and a metal baseplate (Figure 1b).

**FIGURE 1:**
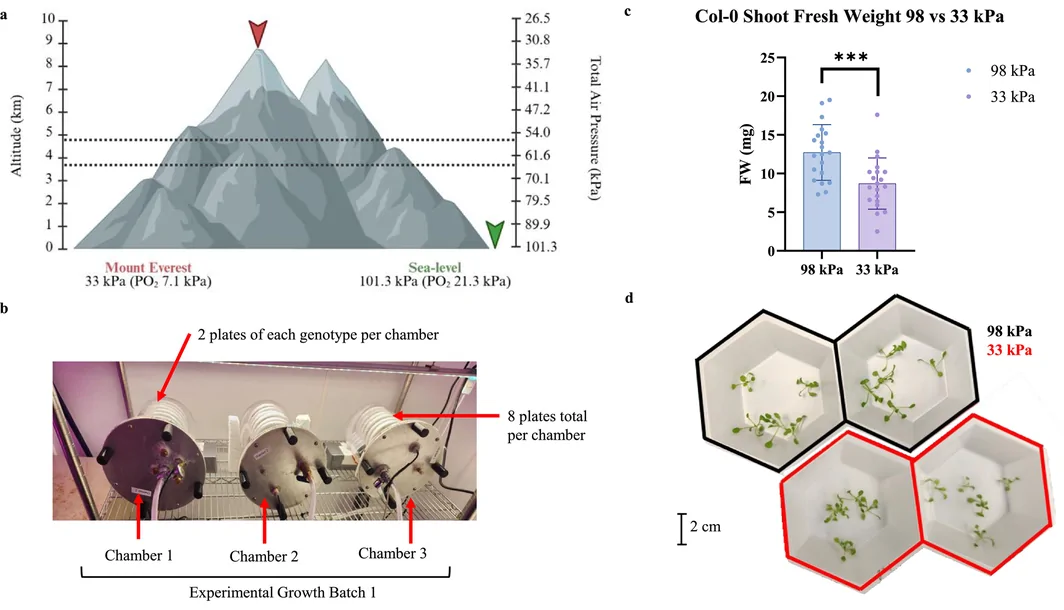
Overview of the pressure chamber setup. A reference graph depicting the inverse relationship between altitude and total atmospheric pressure along with the total atmospheric pressure and partial pressure of oxygen (PO_2_) at notable elevations (a). In addition to the highest and lowest terrestrial atmospheric pressures, indicated in green and red, respectively, the target cabin atmospheric pressure range proposed for future extended duration spaceflight habitats and transit vehicles is denoted by black dashed lines, ~55–65 kPa (Lange et al. 2005). The hypobaric environment is made up of three interconnected acrylic pressure chambers which can run one shared pressure environment across the chambers (b). The chambers have a combined internal volume of ~7500 cm^3^. The operational setup included planted plates in custom racks along with an independent pressure sensor in two of the three chambers. These pressure chambers are connected to a KNF vacuum controller which allows for automatic maintenance of the atmospheric pressure within the vessels. A fresh weight comparison between plants grown 98 and 33 kPa illustrates stunted growth in the plants grown at 33 kPa (c), along with 5 representative plants from each plate grown (d). Panel (a) created in BioRender (Strickland 2025). https://BioRender.com/hb0lc92. Statistical significance testing was completed utilizing an unpaired student's t‐test, *p* < 0.05.

A brief follow‐up experiment was conducted to further investigate the morphological differences observed during the composition‐focused experiments. Two additional plates grown at 33 and 98 kPa in a singular chamber and matching the previous ages were prepared. From each plate, 10 shoots were measured for fresh weights (FWT), yielding 20 individual biological replicates (Figure 1c,d). The chamber relative humidity (RH) was maintained at 80%–90% utilizing moistened cheesecloth swatches to hold moisture and prevent significant water loss from the plant growth media.

### 2.3 Alcohol Insoluble Residue (AIR) Extraction and High‐Performance Anion‐Exchange Chromatography With Pulsed Amperometric Detection (HPAEC‐PAD)

Approximately 100 mg of frozen tissue from each replicate was weighed and placed into a 2‐mL screw cap microfuge tube (Sarstedt, Nümbrecht, Germany). Samples were prepared and analyzed similarly to the methods of Voiniciuc et al. (2015), although the specific procedures are described below. A set of standards utilizing a serial dilution of nine sugars—fucose (Fuc), rhamnose (Rha), arabinose (Ara), galactose (Gal), glucose (Glc), xylose (Xyl), mannose (Man), galacturonic acid (GalA), and glucuronic acid (GlcA)—were used along with an internal standard of 30 μg ribose (Rib). All samples and standards were resuspended from their final dried state using 400 μL of the 30 μg Rib solution. The 100 mg of frozen tissue was allowed to thaw briefly before the addition of 5–6 1 mm zirconium beads. The now thawed samples were homogenized utilizing a Retsch TissueLyser II (Qiagen) using two 24 TissueLyser Adapters (Qiagen) for 1 min at 30 Hz. The adapter was rotated 180° before this process was repeated for a total of three times. The samples then had 1 mL of 70% (v/v) aqueous ethanol added before a short mix using a vortexer. This mixture was then centrifuged for 3 min at 14,000 rpm. This ethanol washing was then repeated. One milliliter of ethanol was then added before utilizing a neoMix thermoshaker (neoLab) to incubate samples at 55°C for 15 min. The mixture was then centrifuged again for 3 min at 14,000 rpm, with the ethanol supernatant removed as waste using a transfer pipette. Then 1 mL of a mixture of 1:1 (v/v) chloroform/methanol was added before agitation utilizing the Retsch mill for 1 min at 30 Hz, followed by repetition after 180° adapter rotation. This mixture was centrifuged for 3 min at 14,000 rpm, and the supernatant was then removed as waste using a transfer pipette. One milliliter of acetone was added to the tube and followed by a short mix with a vortexer (Fisher Vortex Genie 2, Fisher Scientific). The samples were dried using a Techne Dri‐Block DB 3D heater at 60°C while using pressurized air. Approximately 1 mg of the dried alcohol insoluble residue (AIR) was then taken into a new 2 mL screw cap micro tube, and then 300 μL of 2 M trifluoroacetic acid (TFA) was added to each tube. These samples were mixed using the Retsch mill for 1 min at 30 Hz before rotating the adapter 180° and repeating agitation. The capped samples were then hydrolyzed for 90 min at 120°C. The heat blocks were taken out and cooled on ice before sample retrieval. The samples were centrifuged briefly before being dried using pressurized air at 60°C in a fume hood utilizing the dri‐block. These hydrolyzed samples were then resuspended utilizing 400 μL of the 30 μg Rib standard. The monosaccharides released by 2 M trifluoroacetic acid hydrolysis were quantified by high‐performance anion‐exchange chromatography with pulsed amperometric detection (HPAEC‐PAD) on a Metrohm system (model #1.940.1150, Herisau, Switzerland) with a Carb 2 – 250/4.0 column (Mielke et al. 2021). The monosaccharides quantities were normalized using the internal Rib standard and the external standard calibration curve. The results of the analysis were tested for significance utilizing a one‐way ANOVA and a Tukey HSD multiple‐comparison post hoc test in R (v4.4.0).

## 3 Results

### 3.1 Extreme Hypobaria Reduces Shoot Fresh Weight in Arabidopsis

Arabidopsis plants were exposed to a three‐day 33‐kPa pressure treatment beginning at 12 days of age. The relative severity of the pressure treatment is illustrated in Figure 1a, with 33 kPa being roughly the same total pressure found at the peak of Mount Everest. The hardware setup used to execute hypobaric treatments is also documented (Figure 1b). The shoots of Arabidopsis plants exposed to this hypobaric treatment had reductions in fresh weight when compared to the control (Figure 1c). Additional visual examination of the hypobaria exposed shoots illustrated that the plants exposed to 33 kPa appeared smaller and relatively stunted in growth when compared to the 98 kPa control (Figure 1d). Statistical significance testing was completed using an unpaired Student's *t*‐test, with a significance threshold of *p* < 0.05.

### 3.2 Arabidopsis Matrix Polysaccharide Synthesis Is Resilient When Faced With Hypobaric Stress

Despite reductions in fresh weight of Arabidopsis shoots at low pressures (Figure 1c,d), cell wall monosaccharide profiles remained consistent across genotypes and different pressure treatments (Figure 2a,b). In representative shoot examples (Figure 2a), the 98 kPa control was not different from each of the tested hypobaric treatments, ranging from moderate at 75 and 50 kPa to severe hypobaria at 33 kPa. The resilience of cell wall matrix polysaccharides under hypobaric stresses was found in root tissues as well (Tables S1.2–S1.4). There were also no differences in composition between genotypes (Figure 2b). The abundance of all measured matrix polysaccharide monosaccharides was similar across the three experiments, with no consistent differences observed. To examine the reproducibility of each experiment, the monosaccharide quantifications of each experimental repetition for the Col‐0 genotype were compared for both shoot and root tissues at 98 kPa (Figure 3a,b).

**FIGURE 2:**
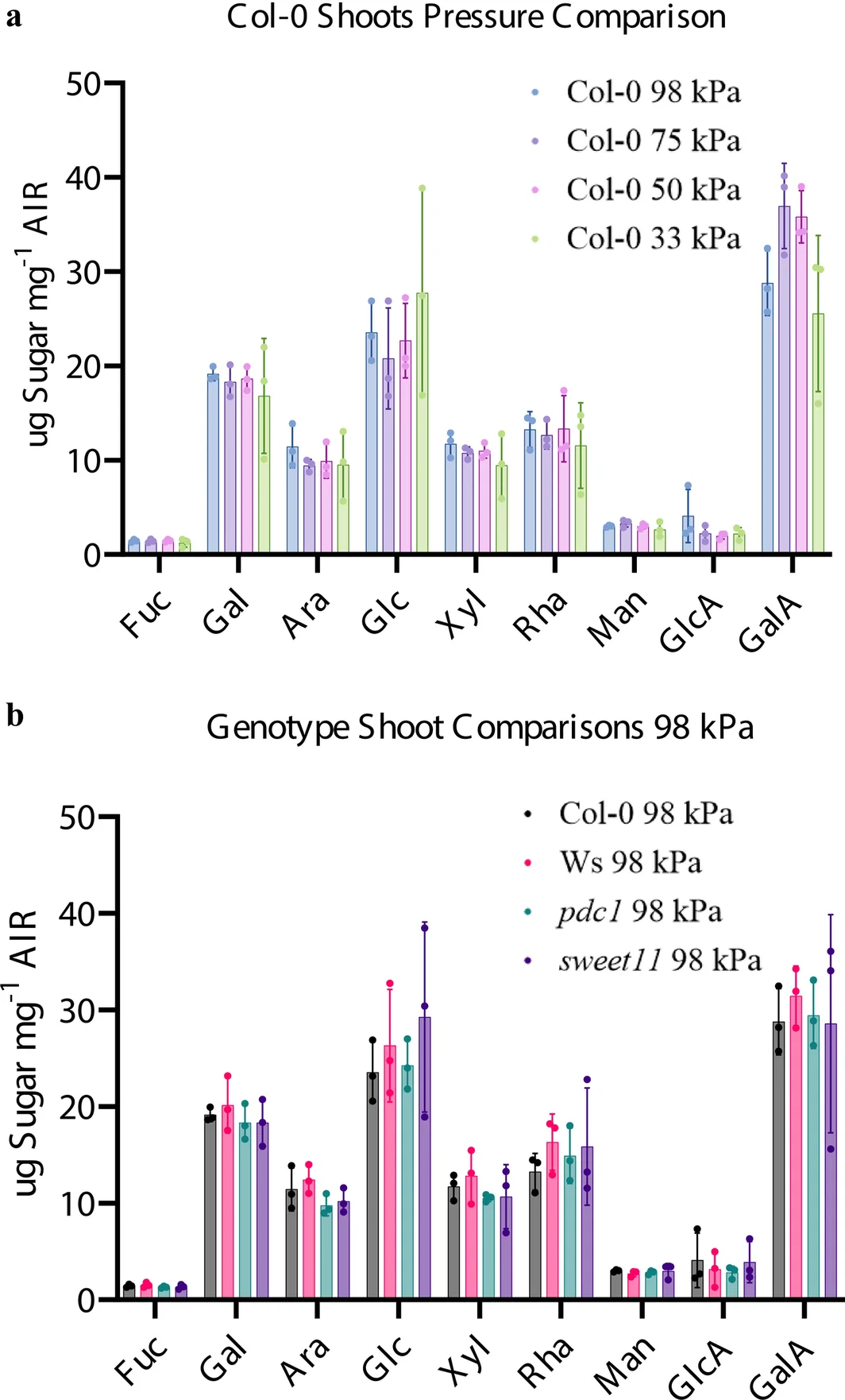
No significant differences due to pressure treatment or ecotype. The figures above illustrate the comparison of the control (98 kPa) atmospheric pressure treatment in shoots to each of the experimental atmospheric pressures (75, 50, and 33 kPa) for one of the three independent repetitions of the experiment, which displayed no consistent, if any, significant differences due to atmospheric pressure (a). A comparison of the genotypes used within the experiment at the control atmospheric pressure treatment also illustrated no significant differences in the matrix polysaccharide composition of the cell wall (b). The bars represent the average of three biological replicates ± standard deviation, with individual sample values marked. Statistical significance was tested using one‐way ANOVA with Tukey HSD post hoc testing. The sample units are provided in μg sugar per mg alcohol insoluble residue (AIR). *p < 0.05.

**FIGURE 3:**
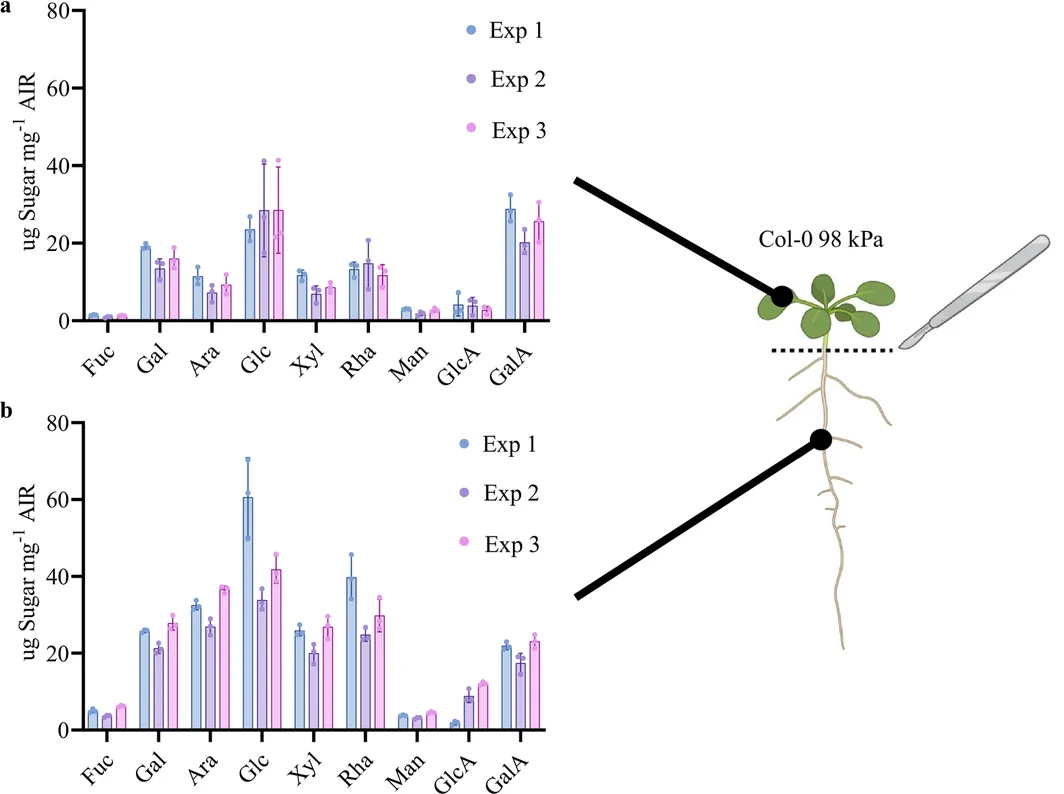
The comparison of experimental values across all three experiments for both tissue types. Sections A and B display the values for shoots and roots of the Col‐0 genotype respectively, at 98 kPa. The comparison displays the variability across the three experimental repetitions, which appears to be slightly larger within roots tissues (b), than shoot tissues (a). The bar charts represent the average of three biological replicates ± standard deviation with individual datapoint values marked. The sample units are provided in μg sugar per mg alcohol insoluble residue (AIR). Created with BioRender (Strickland 2025). https://BioRender.com/w7wiaji.

## 4 Discussion

The objective of the study was to determine if the morphological and transcriptomic data suggesting changes to the plant cell wall composition during hypobaria could be supported with metabolite analyses of cell wall materials from plants exposed to hypobaric environments. Thus, four genotypes of Arabidopsis plants (wild‐type Col‐0 and Ws, *pdc1*, and *sweet11*) were subjected to four atmospheric pressure treatments: 98, 75, 50, and 33 kPa (Figure 1a) in three replicate experiments (Figure 1b). The two wild‐type ecotypes, Ws and Col‐0, provided a direct reference to the WS hypobaric transcriptomes of earlier studies (Paul et al. 2004; Paul et al. 2017; Zhou et al. 2017) and mutants in genes of interest in the widely used Col‐0 background. In all four genotypes and in both roots and shoots, there were no consistent changes to the abundance of cell wall matrix monosaccharides in hypobaric atmospheric pressures (< 98 kPa) (Figure 2a,b and Tables S1.2–S1.5).

Extensive research exists characterizing the effects of hypobaria on plant morphology and biomass ranging across both monocots and dicots (Rajapakse et al. 2009; He et al. 2013; Tang et al. 2015; Guo et al. 2008). In lettuce, growth in hypoxic hypobaric environments of 25 kPa for 10 days results in an approximate 46% decrease in dry leaf weight (Rajapakse et al. 2009). Lettuce grown in normoxic hypobaria displayed a decrease in leaf dry weight of ~65% from the same study (Rajapakse et al. 2009). However, other studies have reported that 15‐day‐old lettuce exposed to 20 days of normoxic hypobaria at 30 kPa did not exhibit a statistically significant reduction in dry weight, despite a trend toward lower dry weight relative to the 101 kPa control (Tang et al. 2015). The often mixed but recurring pattern of nonsignificant reductions in dry biomass, particularly in lettuce, suggests that although hypoxia is likely the primary driver of plant responses under hypoxic hypobaric conditions, hypobaria itself may also contribute to growth responses (Stutte et al. 2022). The general distinctions between hypoxic hypobaria and normoxic hypobaria have been discussed at length (Rajapakse et al. 2009; Paul et al. 2017; Zhou et al. 2017; Tang et al. 2015). In short, these previous studies, including those discussed above, have demonstrated that the effects of hypobaria on plants cannot be attributed solely to the hypoxia that is intrinsic to uncorrected hypobaric atmospheres. For instance, the hypobaric induction of drought metabolisms can initiate downstream stress responses that are independent of hypoxia (Paul et al. 2004). This is exemplified by Arabidopsis grown for 24 h in a 10‐kPa hypoxic/hypobaric environment, which did not exhibit a reduction in fresh weight despite inducing numerous genes associated with drought metabolism, even though water availability was not limiting. Nevertheless, hypoxia remains a major component of the stress imposed by hypobaric exposure.

In the current study, fresh weight was affected when grown in a 33‐kPa hypobaric environment for 3 days (Figure 1c,d). The current findings, together with data from other species, highlight the importance of conducting multi‐day experiments to capture the full morphological response of plants to hypobaria and to establish the effects of severe hypobaric environments on plant biomass. Despite the nonsignificant changes in biomass found previously in Arabidopsis, hundreds of genes have been found to be differentially expressed across multiple total pressure ranges and unique hypobaric atmospheric compositions (Paul et al. 2004, 2017; Paul and Ferl 2006; Zhou et al. 2017).

The Ws ecotype has been previously employed in multiple hypobaric transcriptomic experiments and displayed differential expression of sugar‐metabolism and cell wall–related genes. These genes included sugar will eventually be exported (SWEETs), sucrose synthases (SUSs), galacturonosyltransferases (GAUTs), and xyloglucan endotransglucosylase/hydrolases (XTH) family genes (Paul et al. 2017). The differential expression of genes encoding proteins associated with cell wall (GAUTs and XTHs), and sugar transport (SWEETs, SUSs), suggested possible perturbations to the Arabidopsis cell wall in response to hypobaric exposure. These perturbations could originate either from direct changes to the expression of genes involved in cell wall synthesis and/or degradation or from modification of genes that supply the sugars required for cell wall synthesis. However, this differential expression of cell wall and sugar‐related genes associated with hypobaric exposure did not coincide with a detectable change in cell wall matrix monosaccharide composition in this experiment (Figure 2a and Tables S1.2–S1.5).

To further examine the metabolic consequences of changes in gene expression in response to hypobaria, knockout lines in two genes of interest, *sweet11* and *pdc1*, were used to evaluate the contribution of these genes to the hypobaric response. Both genes were differentially expressed in response to hypobaria in previous studies, with *SWEET11* being repressed and *PDC1* being induced (Paul et al. 2017; Zhou et al. 2017). The *sweet11* mutant was previously reported to have potential differences in cell wall components, specifically in absolute abundance of acidic pectins and rhamnogalacturonan(s), when analyzed using Fourier transform infrared spectroscopy (FTIR) (Le Hir et al. 2015). However, the *sweet11* genotype did not display any consistent, if any, differences in monosaccharide composition from its respective wildtype, Col‐0, or the Ws ecotype at 98 kPa (Figure 2b). Additionally, no consistent differences were found across any of the four tested pressure treatments between *sweet11* and any other geno‐ or eco‐type (Tables S1.2–S1.5).

*PDC1* plays a key role in the plants response to hypoxia (Mithran et al. 2014) and has been previously implicated in Arabidopsis' response to hypobaric environments. *PDC1* saw increases in expression during both normoxic hypobaria, and a range of hypoxic hypobaric environments, 50, 25, 10, and 5 kPa, in Arabidopsis (Paul et al. 2017; Zhou et al. 2017). The differential expression of *PDC1* in both normoxic and hypoxic hypobaric environments illustrates that although *PDC1* does likely play a role in the response to the hypoxic component of hypobaric environments, it is not the sole role of the gene in the physiological adaptation to hypobaric environments (Zhou et al. 2017). In addition, *PDC1* also displayed differential expression in plants of different ages, being consistently differentially expressed in both 5‐ and 10‐day‐old plants illustrating the key role the gene plays in the continued response to hypobaric environments (Zhou et al. 2017). These existing findings made *PDC1* a key gene of interest, and the *pdc1* genotype was employed to determine if *PDC1* was necessary for the resilience of plant cell wall composition to hypoxic hypobaria. The *pdc1* genotype, much like *sweet11*, did not display consistent differences from its respective wildtype, Col‐0, nor any other line employed in the experiment (Figure 2b and Tables S1.2–S1.5).

The current study is the first to employ glycomic techniques to directly investigate how hypoxic, hypobaric environments impact the composition of the plant cell wall matrix at the monosaccharide level. The current findings show that the cell wall matrix in young seedlings is resilient to hypobaric exposure, suggesting that plants can tolerate hypobaric environments without major compositional changes to their structural cell wall components. Although there are no closely comparable datasets available, transcriptomics studies (Paul et al. 2004; Paul et al. 2017; Zhou et al. 2017) provide evidence that genes involved in both cell wall biosynthesis and remodeling are differentially expressed in response to hypobaria. However, these transcriptional changes did not result in detectable differences in plant cell wall matrix composition with respect to monosaccharide abundance in this study. To further verify the conclusions of the analysis presented here the values of the three experimental repetitions from the Col‐0 98 kPa control for both root and shoot tissues are provided to illustrate the similarity of monosaccharide quantification values across experimental repetitions (Figure 3). These inter‐experimental comparisons illustrate the rigor of the overall experiment and further support the current conclusions.

Although Arabidopsis seedling cell walls were resilient to hypobaric exposure, the effects of long‐term (> 7 days) hypobaria on plant cell walls remain unknown. Additional long‐term or lifespan‐encompassing studies may provide further insight into plant adaptation to hypobaric environments. In particular, glycomic analyses of plants germinated and grown in hypobaric environments that more closely reflect future space habitat conditions may help elucidate long‐term changes in cell wall composition or biochemical structure. The results presented here represent only one aspect of the plant cell wall matrix, specifically the overall abundance of its constituent monosaccharides. These findings do not provide information on structural organization or chemical modifications of cell wall polysaccharides that may occur in response to hypobaric exposure. Such changes remain largely unexplored and represent an important avenue for future research.

## Author Contributions

Conceptualization: H.F.S., C.V., R.F. and A.‐L.P. Formal analysis: H.F.S., T.J., C.V., R.F. and A.‐L.P. Funding acquisition: A.‐L.P. and R.F. Investigation: H.F.S., T.J. and G.S. Methodology: C.V., A.C.S., R.F. and A.‐L.P. Resources: C.V. and A.C.S. Writing – original draft: H.F.S. Writing – reviewing and editing: H.F.S., T.J., G.S., C.V., A.C.S., R.F. and A.‐L.P.

## Funding

This work was supported by the National Aeronautics and Space Administration (80NSSC22K0214) and the USDA National Institute of Food and Agriculture, Research Capacity Fund (7004470).

## Conflicts of Interest

The authors declare no conflicts of interest.

## Supporting information


**Table S1:1** T‐DNA insertion screening primers. All primers used to screen *pdc1* and *sweet11* T‐DNA insertion lines for homozygosity are provided.
**Table S1:2:** Cell wall matrix monosaccharide composition data, Experiment 1. Raw and normalized monosaccharide composition values from Experiment 1 are provided for all four genotypes (Col‐0, Ws, *pdc1*, and *sweet11*) across all four pressure treatments (98, 75, 50, and 33 kPa).
**Table S1:3:** Cell wall matrix monosaccharide composition data, Experiment 2. Raw and normalized monosaccharide composition values from Experiment 2 are provided for all four genotypes (Col‐0, Ws, *pdc1*, and *sweet11*) across all four pressure treatments (98, 75, 50, and 33 kPa).
**Table S1:4:** Cell wall matrix monosaccharide composition data, Experiment 3. Raw and normalized monosaccharide composition values from Experiment 3 are provided for all four genotypes (Col‐0, Ws, *pdc1*, and *sweet11*) across all four pressure treatments (98, 75, 50, and 33 kPa).
**Table S1:5:** Statistical testing results for all experimental comparisons. These data include ANOVA results followed by Tukey's HSD post hoc analysis.

## Data Availability

All relevant supplementary data are provided in a single excel file (Supporting Information S1), with individual sheets for specific information or datasets. Table S1.1 contains the genotype screening primers for the T‐DNA insertion lines utilized within the study. Tables S1.2–S1.4 report the raw monosaccharide composition data for Experiments 1 (Table S1.2), 2 (Table S1.3), and 3 (Table S1.4), respectively. Table S1.5 includes the *p*‐values for all statistical tests conducted.
